# Telehealth Versus In-Person Caregiver-Mediated Behavioral Treatment for Challenging Behaviors in Children With Autism Spectrum Disorder: Protocol for COACH (Caregiver Outreach for Autism Coaching at Home) Randomized Controlled Trial

**DOI:** 10.2196/91840

**Published:** 2026-05-26

**Authors:** Joy S Pollard, Leslie S Quiroz, Jennifer A Boyd, Booil Jo, Scott S Hall

**Affiliations:** 1 Behavior Change Institute, Division of Center for Social Dynamics, Albuquerque, NM, United States; 2 Department of Psychiatry and Behavioral Sciences, Division of Child & Adolescent Psychiatry & Child Development, Stanford University School of Medicine, 401 Quarry Road, Room 1365, Stanford, CA, 94305, United States, 1 650-498-4799

**Keywords:** randomized controlled trial, challenging behavior, autism spectrum disorder, telehealth, caregiver-mediated behavioral treatment, aggression, self-injury, practical functional assessment, functional communication training, skill-based treatment

## Abstract

**Background:**

Approximately 40%‐60% of children diagnosed with autism spectrum disorder (ASD) exhibit challenging behaviors such as aggression, elopement, self-injury, and property destruction that can endanger the health and safety of the child or others, often cause significant distress for families, and hinder the child’s developmental progress. Although behavioral treatments grounded in the principles of applied behavior analysis have been shown to effectively reduce or eliminate these behaviors, significant gaps remain in how to deliver these interventions equitably.

**Objective:**

This study aims to compare the efficacy of delivering in-person versus telehealth caregiver-mediated behavioral treatment for challenging behaviors in children with ASD.

**Methods:**

The Caregiver Outreach for Autism Coaching at Home trial is a single-site, multiarm, parallel-group randomized controlled trial to evaluate the efficacy of delivering caregiver-mediated behavioral treatment for children with ASD who exhibit challenging behaviors. We aim to recruit 90 caregiver-child dyads with children with ASD aged 2 years, 0 months to 7 years, 11 months from metropolitan, suburban, and rural areas across New Mexico and the neighboring El Paso, Texas region. Following a practical functional assessment to identify the potential social-environmental variables maintaining the child’s challenging behavior, caregiver-child dyads will be randomized into one of three parallel treatment arms: (1) caregiver-mediated behavioral treatment delivered via telehealth, (2) caregiver-mediated behavioral treatment delivered in person, or (3) caregiver online psychoeducation. Interventions will be delivered by clinicians in weekly 1-hour sessions in the home setting over 12 weeks.

**Results:**

The study was funded in May 2022, and recruitment began in January 2024. As of March 2026, 81 caregiver-child dyads have been enrolled, with equal allocation across the 3 study groups (27 per group). Recruitment is expected to conclude in October 2026, with data analysis planned for Winter 2026 through Spring 2027.

**Conclusions:**

This study will address a critical gap in the scientific literature on scalable, family-centered approaches to safely and effectively reduce challenging behavior in children with ASD and decrease caregiver stress. Findings may inform best practices and payer policy for telehealth-delivered behavioral interventions targeting child safety and caregiver well-being.

## Introduction

### Background

Autism spectrum disorder (ASD) is a neurodevelopmental disorder characterized by deficits in social communication and social interaction that affects approximately 1 in 31 children in the United States [1,2]. Current estimates suggest that children diagnosed with ASD are significantly more likely than their nonautistic peers to exhibit challenging behaviors that pose a safety risk to themselves and others, including aggression, elopement, self-injury, and property destruction [3-5]. Studies estimate that 40%‐60% of children diagnosed with ASD exhibit challenging behaviors, which can vary in intensity and frequency depending on environmental and developmental factors [6]. When effective treatment is not implemented early, these behaviors can escalate and result in serious consequences, including physical restraint, placement in restrictive educational or residential settings, and long-term reliance on sedating medications [7]. Such outcomes can have a lasting impact on the child’s emotional well-being and quality of life, reinforcing the need for timely, compassionate, and evidence-based intervention.

Caregivers of children with ASD also experience heightened stress and emotional strain, alongside increased mental and physical health concerns compared with caregivers of children with other developmental disabilities [8,9]. The demands of managing daily routines, attending multiple therapies, and addressing safety concerns can contribute to significant medical, financial, and social burdens [10,11]. Notably, challenging behaviors and caregiver and family stress are often positively correlated, as greater caregiver stress can exacerbate behavioral difficulties, and persistent behavioral challenges can increase caregiver stress [12-14]. This highlights the importance of addressing the needs of both the child and the family within therapeutic models, as well as engaging caregivers as critical community partners in research, consistent with participatory research approaches that support feasible and family-centered interventions. To support conceptual clarity and alignment with ASD Community Advisory Board feedback, the term “challenging behavior” will be used to describe behaviors such as aggression and self-injury that may pose safety concerns or interfere with learning, communication, or daily routines.

Studies have shown that behavioral interventions grounded in the principles of applied behavior analysis (ABA) can significantly reduce challenging behaviors that may compromise safety or participation in family and community life and simultaneously increase social communication, coping, and adaptive functioning skills [15-17]. Decades of empirical research have led to the recognition of ABA-based treatments as a well-established, evidence-based standard of care endorsed by the US surgeon general, the Centers for Disease Control and Prevention, and the American Academy of Pediatrics [18-20]. Comprehensive ABA treatment plans may include a combination of direct clinician-child sessions, caregiver-mediated components, and interdisciplinary coordination tailored to the child’s individualized needs [21]. Within these models, caregiver-mediated behavioral treatment represents one of several effective approaches for supporting generalization and long-term adaptive functioning. Active caregiver participation can enhance treatment effectiveness and contribute to long-term behavioral outcomes by promoting consistency, reinforcing learned skills, and strengthening family engagement throughout the therapeutic process [22]. Best practices in ABA emphasize that caregiver-mediated interventions are individualized and empower caregivers to navigate their child’s challenging behavior across everyday routines and natural environments [23].

One promising ABA-based intervention for supporting children whose challenging behaviors may impact safety and well-being is skill-based treatment (SBT), a compassionate, trauma-informed behavioral treatment approach grounded in the principles of ABA [24]. SBT involves teaching skill-building steps focused on functional communication, tolerance, and cooperation to support children in developing safer, more adaptive ways to express their needs. SBT is designed to be family-centered, emphasizes rapport building and child assent, and has demonstrated strong feasibility, social validity, and effectiveness across clinic and home settings [24].

Despite strong evidence supporting ABA-based interventions such as SBT, significant gaps remain in how to best deliver these interventions effectively and equitably. Even when efficacious interventions exist, families’ ability to access high-quality, individualized care is often constrained by geographic, socioeconomic, and systemic barriers. These access challenges extend across both rural and urban regions, where families commonly face long waitlists, provider shortages, and financial constraints [25-27]. Addressing these persistent barriers requires the development of scalable, compassionate, and evidence-based care models that can reach families where they live and in the contexts of their daily lives. Given these persistent challenges, researchers and practitioners have increasingly turned to telehealth to improve flexibility, efficiency, and overall equity in service delivery for children with ASD [28,29].

Telehealth uses telecommunication technologies to deliver behavioral treatment in a cost-effective, scalable manner that can reduce these access barriers. Multiple telehealth service delivery models of ABA treatment have been studied, each varying in caregiver involvement and resource requirements. Among these models, caregiver-mediated behavioral treatment via telehealth is a promising approach empowering caregivers with the skills and confidence to implement safe, effective behavioral strategies with their child at home. In this model, a behavioral health provider coaches 1 or more caregivers to implement behavioral strategies with their child via real-time videoconferencing technology (eg, Zoom Workplace version 7.0; Zoom Communications, Inc), eliminating the need for provider travel, minimizing intrusion in the family home, and enabling real-time caregiver support [30,31]. For some families, telehealth may even represent a preferred alternative to in-person service delivery, particularly when the presence of in-person providers is perceived as disruptive to family routines [32,33]. More recently, studies of caregiver-mediated SBT have demonstrated that parents can successfully learn and implement SBT procedures via telehealth to help navigate challenging behaviors in safer, more adaptive ways [34-37].

Although both traditional in-person and telehealth delivery methods have significant research to support their respective effectiveness for decreasing challenging behavior in children with ASD, only 2 studies have directly compared caregiver-mediated behavioral treatment delivered via telehealth versus in person. Lindgren and colleagues [11] conducted a retrospective analysis of 3 separate intervention studies spanning 18 years, examining caregiver-mediated behavioral treatment for children with ASD (aged 2-7 years) to reduce challenging behaviors. In all 3 studies, a functional analysis was conducted to identify the function(s) of the child’s challenging behavior before function-matched behavioral treatments were implemented. In the first study, conducted between 1996 and 2009 (n=44 dyads), a clinician traveled to the family’s home to provide weekly 1-hour treatment sessions in person. In the second study, conducted between 2009 and 2012 (n=20 dyads), families traveled to a regional clinic to receive weekly 1-hour sessions from a remote clinician via telehealth. In the third study, conducted between 2012 and 2014 (n=30 dyads), treatment was delivered via telehealth in weekly 1-hour sessions at the family’s home. Across all 3 cohorts, in-session observations of challenging behavior showed at least an 80% reduction in challenging behaviors from baseline, with telehealth models achieving these outcomes approximately twice as quickly as traditional in-person treatment (mean 9, SD 5.4 weeks vs mean 17, SD 8 weeks). These findings suggest that caregiver-mediated behavioral treatment delivered via telehealth can produce comparable, or even accelerated, treatment outcomes relative to in-person services while reducing the logistical demands on both families and providers.

Marino and colleagues [38] extended this line of research by directly comparing caregiver-mediated behavioral therapy delivered via telehealth with traditional in-person models using a randomized controlled design. Twenty-three parent-child dyads with children diagnosed with ASD, aged 2-10 years, were randomized to receive either 2 hours per week of behavioral treatment delivered via telehealth or in-person caregiver-mediated behavioral treatment over 12 weeks. At the 12-week outcome point, caregiver-child dyads who received treatment via telehealth reported significantly lower levels of parental stress and greater reductions in child challenging behaviors than those who received comparable in-person treatment. Taken together, these findings provide growing evidence that delivering treatments via telehealth offers an effective, cost-effective, and family-centered service delivery modality for ABA-based treatment for children with ASD.

### Objectives

The primary objective of this study is to compare the efficacy of caregiver-mediated behavioral treatment delivered via telehealth versus in-person delivery on 2 key outcomes: levels of child challenging behavior and levels of caregiver stress. Importantly, both treatment modalities will be compared with a caregiver online psychoeducation control. We expect that children who receive caregiver-mediated behavioral treatment delivered via telehealth or in person will demonstrate greater reductions in challenging behavior at the 12-week end point than those who receive the caregiver online psychoeducation control intervention. We also expect that caregivers who receive caregiver-mediated behavioral treatment via telehealth will report lower levels of parental stress associated with their child’s challenging behavior than caregivers who receive the same treatment in person. Secondary objectives of the study will be to examine whether telehealth delivery of caregiver-mediated behavioral treatment offers additional benefits relative to caregiver-mediated behavioral treatment delivered in person. We expect that families who receive caregiver-mediated behavioral treatment delivered via telehealth will demonstrate greater engagement and continuity of care, reflected by more consistent participation and fewer missed sessions than those who receive caregiver-mediated behavioral treatment delivered in person.

### ASD Community Advisory Board

A multidisciplinary ASD community advisory board comprising 11 members contributed directly to the design and planning of this trial. Members were identified based on prior collaboration with the research team on community advisory boards and through referrals from existing advisory members. The board included 6 caregivers, 2 insurance representatives, 2 provider-level clinicians, 1 national trade association representative, and 1 academic researcher external to the study team, with several individuals serving in more than 1 community partner role. The board provided structured feedback on the selection of outcome measures, screening tools, and inclusion and exclusion criteria; reviewed and refined the timing and dosage of treatment sessions; and advised on the functional assessment approach and selected SBT as the behavioral intervention model for the study. Community partners also guided the development of telehealth service delivery procedures and provided recommendations to support caregiver feasibility and safety in home-based settings. In addition, they informed recruitment strategies and contributed to the study acronym, enhancing caregiver accessibility and engagement. Their involvement ensured that the protocol was family-centered, clinically feasible, and responsive to the needs and priorities of families and service providers.

### Trial Design

The Caregiver Outreach Autism Coaching at Home study is a single-site, multiarm, parallel-group, randomized controlled superiority trial targeted to decrease challenging behaviors in children with ASD. We aim to recruit 90 caregiver-child dyads to participate in the 4-year study. Each dyad will first receive a practical functional assessment (PFA) to identify the function(s) of the child’s challenging behavior and subsequently randomized (1:1:1) to telehealth-delivered SBT, in-person SBT, or online psychoeducation.

**Figure 1.**
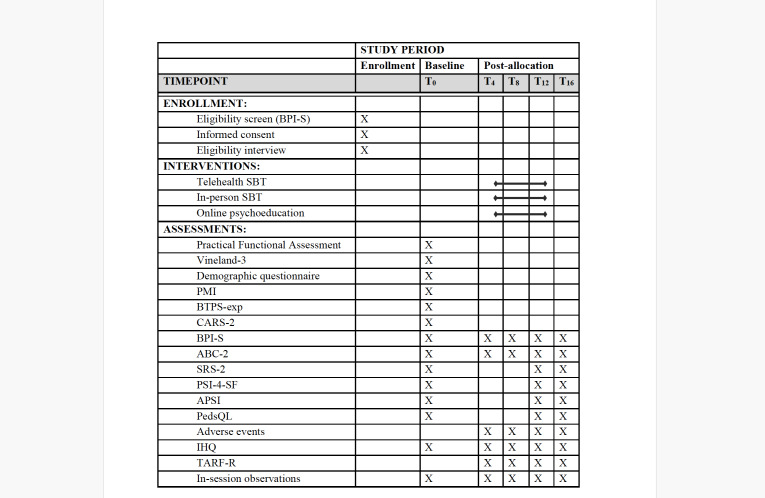
SPIRIT (Standard Protocol Items: Recommendations for Interventional Trials) diagram of enrollment, interventions, and assessments. ABC-2: Aberrant Behavior Checklist—Second Edition; APSI: Autism Parenting Stress Index; BPI-S: Behavior Problems Inventory—Short Form; BTPS-exp: Barriers to Treatment Participation Scale—Expectancies; CARS-2: Childhood Autism Rating Scale, 2nd Edition; IHQ: Intervention History Questionnaire; PedsQL: Pediatric Quality of Life Family Impact Module; PMI: Parent Motivation Inventory; PSI-4-SF: Parenting Stress Index-Fourth Edition—Short Form; SBT: skill-based treatment; SRS-2: Social Responsiveness Scale—2nd Edition; TARF-R: Treatment Acceptability Rating Form—Revised.

**Figure 2.**
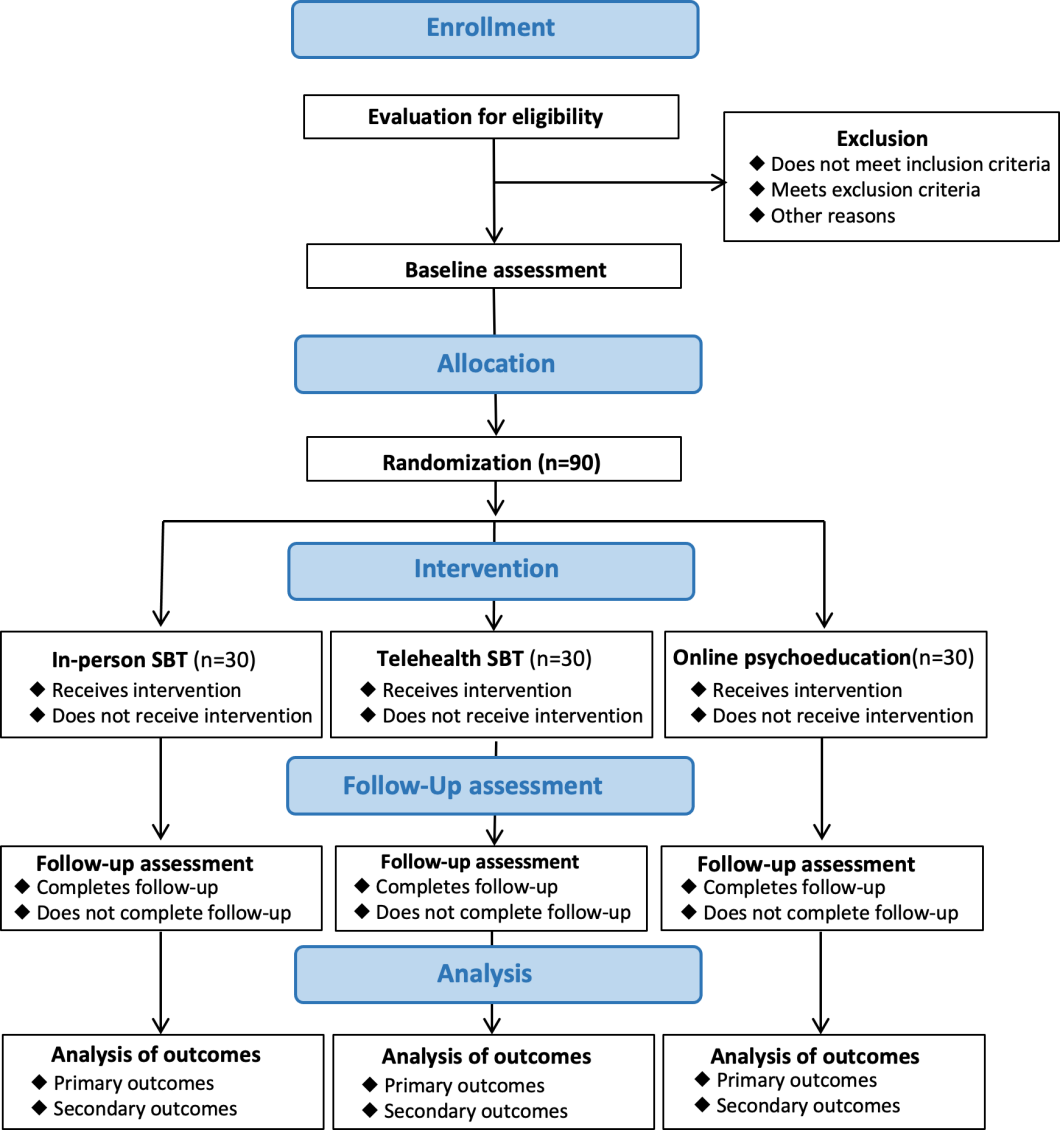
Flow diagram of the COACH (Caregiver Outreach for Autism Coaching at Home) trial.

The primary outcome will be in-session rates of challenging behavior observed at the 12-week outcome point, measured through standardized behavioral observation procedures as well as reported levels of caregiver stress. Secondary outcomes will include family quality of life, treatment satisfaction, and indicators of sustained family engagement and continuity of care, reflected by the number of scheduled sessions completed, missed appointments, and participant attrition at the 12-week outcome (end of treatment). A SPIRIT (Standard Protocol Items: Recommendations for Interventional Trials) diagram showing the schedule of enrollment, interventions, and assessments is shown in he overall study is shown in Figure 1. The study flowchart is shown in Figure 2. For all participants, in-session observations and parent questionnaires will be conducted at 5 assessment points: baseline (T0), 4 weeks (T4), 8 weeks (T8), 12 weeks (T12), and 16 weeks (T16). The study protocol (version 1.9; May 8, 2025) follows the SPIRIT statement. The trial has been registered at ClinicalTrials.gov (identifier: NCT05268796). All methods were finalized before enrollment began. Subsequent protocol revisions included decreasing the lower bound of the age range to include children aged 2 years and including additional rural counties in New Mexico to increase the reach of the study.

## Methods

### Study Setting

All treatment procedures will be conducted in the family home located within 19 metropolitan, suburban, and rural counties of New Mexico depicted in , and within 2 counties in Northwest Texas (El Paso and Hudspeth), given their proximity to the state border of New Mexico.

### Eligibility Criteria

#### Child Inclusion Criteria

Caregiver-child dyads will be eligible to participate if the child is between 2 years, 0 months and 7 years, 11 months of age and has a documented medical or educational diagnosis of ASD. Children will be included if they are reported to exhibit at least 1 form of daily or hourly self-injurious or aggressive behavior on the Behavior Problems Inventory—Short Form (BPI-S) [39,40] and live at home with their caregiver.

#### Child Exclusion Criteria

Children will be excluded if they engage in behavior that may inflict moderate to severe damage to themselves or to others (eg, biting through the skin, eye gouging, fracturing bones, and significant property damage) requiring any level of medical attention. Children will also be excluded if they have a concomitant medical or genetic condition that, in the judgment of the principal investigator (SH , would prevent them from safely participating in or benefiting from the behavioral intervention, or if they have an underlying medical condition that is better treated through medical rather than behavioral care.

#### Caregiver Inclusion Criteria

Eligible caregivers will be ages 21 years or older, be comfortable speaking and reading in English, and be available to receive coaching for 1 hour per week over 12 weeks, between 9 AM and 7 PM, Monday through Saturday. Caregivers will also be willing to adhere to the study intervention regimen, allow a clinician to visit their home for 1 hour per week for 12 weeks (if randomized to the in-person group), and provide consent for video recording of sessions.

#### Caregiver Exclusion Criteria

Caregivers will also be excluded if they are receiving direct coaching to manage their child’s challenging behaviors, have activity restrictions that limit their ability to respond to those behaviors, or have already participated in the study with another child. Clinicians who deliver the interventions will be registered as a Board Certified Behavior Analyst (BCBA) or a Board Certified Behavior Analyst—Doctoral (BCBA-D) with at least 1 year of postcertification clinical experience providing behavioral assessment and intervention for children with ASD. All clinicians will be credentialed at level 1 in SBT procedures (FTF Behavioral Consulting) and demonstrate SBT implementation integrity of ≥90% on a fidelity checklist during pilot sessions before working with study participants. Before initiating any participant sessions, clinicians will also complete study-specific training on trial procedures, data collection, and telehealth delivery standards.

### Screening and Recruitment Procedures

Caregivers who express interest in the study will complete a brief REDCap (Research Electronic Data Capture) screening survey to determine preliminary eligibility. The survey will collect demographic information about the caregiver-child dyad and include the BPI-S to screen for behaviors that pose a safety risk. Families who meet preliminary eligibility criteria will be contacted by a research assistant to schedule a 20-minute phone interview to confirm eligibility. During this call, the research assistant will complete the Functional Analysis Screening Tool (FAST) [41] to gather additional information about the factors that may influence the child’s challenging behaviors. If a family does not meet the eligibility criteria, they will be informed and offered a referral to ABA treatment. If the research assistant has questions regarding family eligibility or underlying medical concerns, they will present the information at weekly meetings for review.

### Ethical Considerations

Ethics approval was obtained from Stanford University Institutional Review Board (IRB) (protocol number 70567) and WIRB-Copernicus Group Independent Review Board (protocol number 20221079). Written informed consent will be obtained from caregivers before participation in any study activities. Caregivers will provide consent to video recording of sessions for treatment fidelity monitoring, behavioral coding, and data analysis. Any future use of recordings for educational or dissemination purposes will require a separate optional consent process. Confidentiality safeguards are described in the “Data Management” section.

### Baseline Data Collection

The measures collected at baseline (T0) to characterize participant demographics will include autism symptom severity, social and adaptive functioning, caregiver motivation, and perceived barriers to participation. Following completion of these assessments, a PFA will be conducted with all participants to experimentally confirm the functions(s) of the challenging behaviors identified in the caregiver interview (see the “Outcomes” section).

### Procedures for Assessments and Intervention Preparation

#### Technology Requirements

All remote assessments and telehealth-delivered intervention sessions will be conducted using Zoom. Families must have access to a stable internet connection capable of supporting synchronous videoconferencing. Zoom waiting rooms, passcodes, and authenticated entry will be required to ensure secure access to sessions.

To promote consistency and reduce technology-related barriers, each family will receive a standardized technology kit. The kit includes a 9th-generation iPad with an Otterbox protective case, an iPad charger, an Arkon TAB086-12 heavy-duty adjustable stand for optimal camera positioning, a Bluetooth earbud for caregiver-clinician communication, and written setup instructions. These materials are intended to ensure adequate video and audio quality across telehealth sessions.

During the initial call, clinicians will coach caregivers on configuring the device, accessing Zoom, optimizing camera placement, and ensuring clear audio transmission. If technical difficulties arise during a session, such as connectivity issues, audio disruptions, or video freezing, clinicians will follow a predefined troubleshooting protocol, which may include reconnecting through Zoom, contacting the caregiver by phone, or rescheduling the session if needed. Families will be provided with contact information for technical support and instructions for requesting replacement equipment in cases of malfunction (eg, damaged stand, lost earbud, or nonfunctional iPad). Clinicians delivering telehealth sessions will receive training in telehealth best practices, including platform navigation, camera optimization, privacy safeguards, and remote coaching strategies, to promote competent and consistent delivery of telehealth-based procedures.

### Environment and Safety Planning

Before initiating any assessment or intervention procedures, clinicians will coach caregivers on preparing a safe and functional treatment environment within the home. The designated session space will be required to measure approximately 6×6 feet, be free of breakable or hazardous items, allow for controlled access to exits when feasible, and include materials necessary for the safe and consistent delivery of all study procedures. Caregivers will receive guidance on arranging the area to minimize distractions, optimize camera visibility, and support safe, high-fidelity implementation of all study procedures across all treatment arms.

When children exhibit behaviors that may compromise safety, clinicians will assist caregivers in implementing simple, practical environmental arrangements that can be applied reliably during both assessment and treatment sessions. Examples will include securing long hair, wearing long sleeves, using cushions or pillows as a soft barrier, and temporarily removing breakable items or objects that could pose a hazard if thrown. These precautions will help ensure a safe and predictable environment throughout all study procedures.

### Practical Functional Assessment

The PFA will involve two main components: (1) conducting an open-ended interview with the caregiver and (2) a performance-based interview-informed synthesized contingency analysis (IISCA) with the caregiver and the child to experimentally confirm the potential variables maintaining challenging behavior identified in the interview. The open-ended interview will be conducted over synchronous videoconferencing via Zoom or by phone, which will be scheduled for approximately 1 hour. During the interview, the clinician will gather information about the child’s personally relevant reinforcers, the antecedents and consequences that typically surround the child’s dangerous and nondangerous behavior, and the topography of those behaviors [42-44]. Additional questions will be included to determine an appropriate therapeutic space, the placement of cameras for video recording sessions, and information about typical parent-child interactions. Finally, the clinician will review an environmental safety checklist to assist the caregiver in preparing the session space for the performance-based IISCA.

The performance-based IISCA will be conducted via telehealth with all participants [45,46]. To ensure the safety of the child and caregiver, the clinician will coach the caregiver on the performance-based IISCA procedures using behavioral skills training [47]. The clinician will review PowerPoint slides with the caregiver on how to (1) provide synthesized reinforcement (SR) to their child, (2) present evocative conditions to their child, and (3) reinforce the first instance of challenging behavior [48]. The clinician will review the operational definitions of challenging behaviors and note any behavioral topographies not identified during the open-ended interview or on the BPI-S. The clinician will ensure caregiver competency and confidence with the protocol by modeling and practicing all steps with the caregiver before implementation with the child.

The performance-based IISCA will consist of 2 blocks of 5 trials, alternating between SR and establishing operation (EO) intervals. In each block, the analysis will begin with caregivers allowing their child to play with their favorite toys for a minimum of 4 minutes. Following the SR interval, the caregiver will be coached to present an EO interval. The EO will consist of a sequence of predictable, stepwise cues signaling the end of reinforcement (eg, standing, clapping, moving closer to the child, and saying, “all done play”). If the child engages in challenging behavior at any point in the sequence, the caregiver will be coached to rapidly remove all evocative events and provide the child with SR for at least 30 seconds. No consequences will be provided for challenging behavior during the SR intervals. Instead, the clinician will coach the parent to help the child minimize evocative events (eg, by limiting questions, directing play, and providing choices) and provide additional reinforcing materials for the child. If at any point during a session the child exhibits challenging behavior that poses a safety risk, or the caregiver expresses discomfort, the analysis will be paused until the child returns to engaging in play for a minimum of 4 minutes.

The rate of challenging behavior observed in the EO and SR intervals will be plotted across trials to identify the evocative condition(s) that most reliably trigger the child’s challenging behavior. To aid visual inspection of the data, Automated Nonparametric Statistical Analysis will be conducted to confirm differentiation between the EO and SR intervals [49]. The clinician will then develop the appropriate SBT protocol based on the outcome of the IISCA. Clinicians will also score the child’s language level on a 4-point scale (1=nonverbal, 2=1-word utterances, 3=short disfluent sentences, and 4=full fluency).

### Interventions

Following completion of the PFA, participants will be randomized to one of three interventions: (1) in-person SBT, (2) telehealth-delivered SBT, and (3) caregiver online psychoeducation. Interventions will be delivered in 1-hour sessions once per week over 12 weeks.

### Intervention Materials

To support standardized implementation of the intervention procedures, families randomized to either in-person SBT or telehealth-delivered SBT will receive a set of intervention materials including a laminated 3×3 inches functional communication card labeled “my way,” and an 8½×11 inches laminated stimulus board with a Velcro strip. These materials will be introduced and modeled by clinicians during early sessions, and caregivers will be coached on their appropriate use during treatment implementation.

### In-Person SBT

For participants assigned to receive in-person SBT, a trained clinician will travel to the family home each week to coach the caregiver to implement SBT with their child in person. All therapeutic procedures will follow the standardized SBT protocol described in the “Phase 1” and “Phase 2” sections. Clinicians will directly assess and arrange the environment, provide in-person modeling for caregivers, and offer on-site support for behavioral management and safety when indicated. The clinician will begin the session by reviewing the current step in the treatment protocol, determining whether external factors may impact behavior (eg, illness and lack of sleep), and practicing previous steps with the caregiver as needed. Treatment will include two main phases: (1) functional communication training (FCT) and (2) contextually appropriate behavior (CAB) training.

#### Phase 1: Functional Communication Training

In phase 1, caregivers will be coached to implement FCT with their child to decrease their child’s challenging behavior and simultaneously help their child gain access to their personalized reinforcers appropriately. Treatment will begin by teaching the child to emit a simple functional communication response (sFCR) (eg, “my way” or “my turn”). On successive trials, caregivers will present their child’s individualized EO identified in the PFA at baseline using the standardized sequence outlined in the “PFA” section and then prompt the child to emit the sFCR. Prompting procedures will be tailored to the child using most-to-least prompting. Following a correct response, the caregiver will be coached to return the child to SR (eg, the caregiver will say “Ok, you can have your way”).

Once the child reliably emits the sFCR with 100% accuracy, a more complex functional communication response (eg, “my way, please” or “my turn please”) will be introduced to build tolerance for delayed or denied reinforcement. The FCT phase will be complete once the child emits the complex functional communication response reliably with 100% correct responding across 5 trials.

#### Phase 2: CAB Training

In phase 2, caregivers will be coached to teach their child a progressive sequence of CABs of increasing complexity and difficulty, including (1) relinquishing reinforcement (initial CAB), (2) transitioning between activities (intermediate CAB), and (3) cooperating with adult instructions (terminal CAB). Consistent with best practice guidelines for SBT, the targeted communication and cooperation skills will incorporate typical daily routines (eg, cleaning up and completing academic work) that caregivers identify as contexts in which challenging behavior commonly occurs.

Each 5-trial block in this phase will consist of 2 FCT trials and 3 CAB trials presented in randomized order to minimize predictability and maintain the child’s communication responses. During CAB trials, the caregiver will be coached to present an individualized EO in the progressive sequence and prompt their child to exhibit the targeted CAB response. All CABs will be taught using 3-step least-to-most prompting (ie, “Tell them,” “Show them,” and “Help them”) [48]. The child will progress to the next step in the CAB chain only after demonstrating 100% correct responding within a 5-trial block. Clinicians will adjust prompting intensity or pacing based on performance, but the structure and sequence of SBT procedures will remain constant across participants. A 5-trial block structure will be used throughout treatment. Because treatment progression is based on individual performance, participants may reach different phases by week 12.

### Telehealth-Delivered SBT

Caregivers assigned to the telehealth-delivered SBT arm will be coached remotely by the BCBA clinician via Zoom to implement SBT using the methods described by Quiroz et al [34] and Metras et al [35]. Remote behavioral skills training will be provided using didactic instruction, role-play–based modeling, rehearsal, and feedback. Because clinicians cannot model procedures directly with their child, they will provide remote demonstration strategies (eg, gesture modeling, role-play, and screen-shared examples). Safety procedures will rely on caregiver-directed environmental modification, guided verbally by the clinician as needed. All procedures will follow the standardized SBT protocol described in the In-Person SBT section.

### Caregiver Online Psychoeducation

Caregivers randomized to receive caregiver online psychoeducation will be assigned self-paced online training modules from the Autism Distance Education Parent Training program. The program will be implemented in 2 modules over the 12-week intervention period. Module 1 titled *strategies for teaching functional skills* comprises 10 lessons that describe ABA principles such as contingencies, antecedent planning, reinforcement, prompting, chaining, task analysis, and error correction. Module 2 titled *positive behavior strategies for your child* comprises 10 lessons that review specific strategies to understand, prevent, and respond to a child’s challenging behaviors.

Caregivers will be provided with an iPad and sent weekly links to the asynchronous lessons. These self-paced lessons provide behavioral techniques developed from ABA and include multimedia presentations, video demonstrations, interactive activities, a glossary of terms, guided notes, self-assessment quizzes, and homework assignments. Each lesson lasts approximately 15‐30 minutes, and caregivers will be asked to complete 1‐2 lessons each week.

In addition to weekly asynchronous lessons, the clinician will schedule weekly 30-minute caregiver meetings over Zoom, without their child present. The clinician will provide PowerPoint slides specific to the assigned material, discuss assignments, and clarify questions. These discussions will reinforce conceptual understanding but will not include direct coaching with the child present or in vivo feedback. Each session will therefore consist of approximately 30 minutes of self-paced modules completed in advance plus a 30-minute discussion session with the clinician.

During weeks 4, 8, 12, and 16, caregivers will be coached to deliver the EO that was identified in their child’s performance-based IISCA and to use the strategies that they learned in the online modules. These probe sessions will be designed to assess whether the child is acquiring the specific communication and cooperation skills targeted in the Autism Distance Education Parent Training curriculum. If the child displays any signs of escalation of challenging behavior during the EO, the caregiver will be coached to remove the EO immediately and provide SR. If the child engages in any of the targeted appropriate behaviors (FCR and cooperation) with or without prompting, the parent will be coached to provide access to reinforcement, if needed. If the child does not cooperate with the directive and does not engage in challenging behaviors, the clinician will coach the parent to gradually progress the EO until the child engages in targeted appropriate behavior (ie, FCR or cooperation) or until challenging behavior occurs.

#### Criteria for Discontinuing or Modifying Allocated Interventions

Participants may withdraw from the study at any time. Investigators may discontinue a participant for (1) nonparticipation with study procedures, (2) inability to contact the family, and (3) any event, medical condition, or situation occurs such that continued data collection would not be in the participant’s best interest or may confound outcomes. For participants who remain enrolled, intervention procedures may be modified within predefined clinical parameters to ensure participant safety, accommodate individual learning needs, or maintain engagement. Consistent with the SBT framework, clinicians may adjust session pacing, reinforcement schedules, prompting procedures, visual supports, or task demands based on participant progress and caregiver feedback. For telehealth sessions, coaching methods may be tailored to caregivers’ comfort and logistical constraints. For example, the clinician may schedule brief practice segments before beginning the formal sessions with the child or provide written guidance. The targeted functional relations, the sequence of SBT phases, and the treatment dose will not be altered. Any changes that substitute non-SBT methods (eg, punishment-based procedures) will be prohibited.

If a behavior escalates to a level that compromises safety, the clinician will pause skills training and ensure that the participant returns to HRE before resuming intervention. All modifications will be reviewed in weekly supervision meetings and documented in session notes to ensure adherence to the study protocol and core SBT components.

#### Strategies to Improve Adherence

To support consistent participation and maintain treatment fidelity across the 12-week intervention period, multiple strategies will be used to promote caregiver engagement. Automated calendar reminders will be sent prior to each scheduled session. Sessions will be scheduled at a consistent, convenient time of day for the family (eg, late afternoon or early evening) to enhance predictability and reduce scheduling barriers.

Each session will begin with a brief 5-minute check-in to review recent events that may affect participation and will conclude with a 10-minute debrief focused on answering caregiver questions and planning between-session practice. Caregivers will receive clear guidance for practicing intervention strategies outside of sessions and will be encouraged to integrate brief practice opportunities into daily routines at least 1‐2 times per day. Clinicians will complete a brief postsession form documenting session duration, travel time (if applicable), caregiver engagement, reported out-of-session practice, and any contextual factors that may influence adherence. This information will be reviewed in weekly supervision meetings to proactively identify barriers to participation and offer tailored support to families as needed.

### Relevant Concomitant Care Permitted or Prohibited

Participants may continue with concurrent educational, medical, or allied health services (eg, speech or occupational therapy) during participation. Medication usage will be assessed at each time point and documented in REDCap. To prevent confounding of study outcomes, caregivers will be asked to refrain from starting other behavioral parent training for intensive behavior reduction programs during the 12-week intervention. All concurrent therapies will be documented using the Intervention History Questionnaire.

### Posttrial Care

At any time during participation, the family will not be prevented from receiving direct service ABA treatment. Upon request and with consent, results from the study may be shared with the family’s provider to support transition to ongoing care. After trial completion, all families, including those in the online psychoeducation arm, will be offered referrals to qualified ABA providers in their region. The interventions included in this study are low-risk; however, should any participant experience harm or distress related to study procedures, clinicians will provide appropriate support and facilitate a referral to appropriate care.

### Outcomes

#### Primary Outcomes

*In-session rate of challenging behavior*: The primary outcome in this trial will be the rate of in-session challenging behavior, defined as the number of occurrences of challenging behavior per minute observed across all trials in a session. Challenging behavior will be categorized as either *dangerous* (ie, behaviors that have the potential to cause harm to the child or others such as hitting, kicking, scratching, and throwing items at the person) or *nondangerous* (ie, behaviors that do not have the potential to cause harm to the child or others but have been identified as precursors to dangerous behaviors such as whining, dropping to the floor, and elopement). The specific behavioral topographies for each child will be identified through the caregiver screening and confirmed during the initial performance-based IISCA assessment. Data will be collected during monthly probe sessions at baseline, weeks 4, 8, and 12, and at week 16 (4-week posttreatment follow-up). The primary analysis will examine the change in rate across baseline, week 4, week 8, and week 12 (end of active treatment).

*Parenting Stress Index—Fourth Edition* Short Form (PSI-4-SF) [50]: The PSI-4-SF is a 36-item instrument measuring parent stress in 3 domains: Parental Distress, Parent-Child Dysfunctional Interaction, and Difficult Child. The primary analysis will examine change in total stress from baseline to week 12 and at week 16 (4-week posttreatment follow-up).

#### Secondary Outcomes

Secondary outcomes will include caregiver-report measures of challenging behavior, quality of life, concurrent interventions, treatment satisfaction, and adverse events (AEs). All measures will be assessed at baseline and at weeks 4, 8, 12, and 16, except the Treatment Acceptability Rating Form–Revised (TARF-R), which will be administered at weeks 4, 8, 12, and 16.

### Measures

*Behavior Problems Inventory—Short Form* [39,40]: The BPI-S is a 30-item caregiver questionnaire that assesses self-injurious behavior (7 items), aggressive and destructive behavior (11 items), and stereotyped behavior (12 items). Items are scored on a 5-point frequency scale (never, monthly, weekly, daily, and hourly) and on a 4-point severity scale (no problem, mild, moderate, and severe).

*Aberrant Behavior Checklist—Second Edition* [51]: The Aberrant Behavior Checklist—Second Edition is a 58-item scale assessing behavior severity across 5 subscales: Irritability, Social Withdrawal, Stereotypy, Hyperactivity, and Inappropriate Speech*.* Percent reduction in the Irritability subscale from baseline to week 12 will be the primary metric, with a ≥25% reduction considered clinically meaningful.

*Autism Parenting Stress Index* (APSI) [52]: The APSI is a 13-item questionnaire that assesses parenting stress across communication, socialization, adaptive living skills, and maladaptive behavior domains. Change in total score from baseline to week 12 will be examined.

*Pediatric Quality of Life (PedsQL) Family Impact Module* [53]: The PedsQL is a 36-item standardized instrument that measures the impact of chronic pediatric health conditions on caregivers and families across 8 dimensions: physical functioning, emotional functioning, social functioning, cognitive functioning, communication, worry, daily activities, and family relationships.

*Intervention History Questionnaire*: The Intervention History Questionnaire will be used to document concurrent therapies (eg, number of hours spent in speech and occupational therapy per week) and the type, dosage, and time of administration of any psychotropic medications.

*Treatment Acceptability Rating Form—Revised* [54]: The TARF-R is a 21-item caregiver satisfaction scale with 6 subscales: Willingness, Reasonableness, Effectiveness, Cost, Side effects, and Disruptiveness.

*AE checklist*: The clinician will complete an AE checklist in REDCap to capture the occurrence, severity, and attribution of any adverse or unintended events.

### Additional Measures

Baseline measures will be summarized descriptively (means, SDs, and frequencies) to characterize the sample and, for adaptive and social-behavioral functioning measures (Vineland Adaptive Behavior Scales, Third Edition [Vineland-3], Social Responsiveness Scale, Second Edition [SRS-2], and Childhood Autism Rating Scale, 2nd Edition [CARS-2]), examined poststudy to confirm baseline comparability across groups following randomization:

*Demographic Questionnaire*: The Demographic Questionnaire is designed to assess the child’s age, diagnosis, and location; child or caregiver vision or hearing impairments; family availability; caregiver language proficiency; and family demographics (ethnicity, educational levels, marital status, income levels, and household composition). These data will be used to describe the study sample and will inform covariate analyses.

*Childhood Autism Rating Scale, 2nd Edition* [55]: The CARS-2 is a 15-item observational rating scale designed to screen for autism symptoms for children aged 2 years and older. CARS-2 scores will be used to describe autism symptom severity within the sample and may be included as a covariate in exploratory analyses to adjust for baseline differences in symptom severity.

*Social Responsiveness Scale, Second Edition* [56]: The SRS-2 is a 65-item rating scale that assesses autism-related social behavior in individuals aged 30 months to 89 years. Items are divided into five treatment subscales: (1) Social Awareness, (2) Social Cognition, (3) Social Communication, (4) Social Motivation, and (5) Restricted Interests and Repetitive Behavior. The primary measurement variable will be the total raw score, which will be analyzed descriptively to characterize the sample’s social-behavioral functioning.

*Vineland-3* [57]: The Vineland-3 measures adaptive behavior via caregiver report across communication, daily living skills, and socialization, with 2 optional domains (motor skills and maladaptive behaviors). The Adaptive Behavior Composite and domain standard scores will be analyzed descriptively to characterize baseline adaptive functioning and may be used as covariates in exploratory analyses.

*Parent Motivation Inventory* (PMI) [58]: The PMI is a 25-item questionnaire measuring parent motivation to participate in treatment. It evaluates the desire for change in their child, the willingness to change their own behaviors to influence the child’s change, and the perceived ability to change those behaviors. The total PMI score will be used to index caregiver motivation at baseline. Secondary exploratory analyses will examine whether baseline caregiver motivation predicts treatment engagement and outcomes, including reductions in caregiver stress and improvements in behavior.

*Barriers to Treatment Participation Scale—Expectancies* (BTPS-exp) [59]: The BTPS-exp is a 44-item questionnaire that assesses perceived treatment barriers across 4 primary areas: stressors or obstacles that compete with treatment, treatment demands and issues, perceived relevance of treatment, and the caregiver-therapist relationship. Total and subscale scores will describe perceived barriers to treatment at baseline. Secondary exploratory analyses will examine whether baseline BTPS-exp scores predict treatment outcomes, including reductions in behavior and caregiver stress and treatment satisfaction.

### Participant Timeline

Recruitment of clinical trial participants began in January 2024 and is scheduled for completion in October 2026. We aim to enroll 10 caregiver-child dyads per quarter who meet all study eligibility criteria. Each family will participate in the trial for a total of 16 weeks, consisting of 12 weeks of active treatment followed by a 4-week posttreatment follow-up.

### Sample Size

The sample size will be 90 caregiver-child dyads, with each dyad randomized to one of three arms: (1) telehealth-delivered SBT (N_1_=30), (2) in-person SBT (N_2_=30), and (3) caregiver online psychoeducation (N_3_=30). Power estimation was based on the primary outcome, the rate of challenging behavior recorded during in-session observations. Power analyses were conducted using mixed-effects modeling, accounting for anticipated attrition of 20% by week 12. Therefore, oversampling beyond the initial total of 90 caregiver-child dyads is not planned.

Preliminary data from our prior study demonstrated a large intervention effect (Cohen *d*=1.5) on the rate of challenging behavior during in-session observations after a 12-week comparable intervention delivered via telehealth [59]. To provide a conservative estimate, we used an effect size of *d*=0.8 for our power estimation. We conservatively assumed an intraclass correlation across repeated measures of 0.7 based on the established test-retest reliability of 0.8‐0.9 for in-session observations of challenging behavior. Under these assumptions, with initial N=30 per group and 20% attrition, the estimated power to detect a significant intervention effect is 0.88 (α=.05, 2-tailed).

We further hypothesize that parenting stress, as measured by the PSI-4-SF, will be significantly lower at the 12-week assessment point among caregivers receiving telehealth-delivered SBT compared with those receiving in-person SBT. As a clinically meaningful minimum effect size, we assumed an effect size (Cohen’s *d*=0.8), which is also supported by previous studies. We assumed a 20% attrition rate by 12 weeks. Under this scenario, the estimated power to detect a group difference is 80% (2-tailed, α=.05) based on a simple group comparison. Power is expected to improve when a longitudinal mixed-effects model incorporating repeated measures is applied.

We will examine caregivers’ implementation fidelity as a potential mediator of reductions in caregiver stress. In the absence of prior data, we assume a medium effect size for the treatment effect on fidelity (eligibility criteria for mediation), yielding 80% power with an initial sample of N=90 dyads and 20% attrition. For the second part (analytical criteria for mediation), we assumed a medium correlation (*r*=0.3) between caregiver fidelity and levels of caregiver stress, which leads to ample power to detect mediation (power=0.96; 2-tailed, α=.05). Again, we will gain power as we conduct the mediation analysis in our mixed-effects modeling framework. Finally, exploratory moderator analyses will emphasize hypothesis generation and clinical significance (effect size) rather than reliance on statistical significance testing (ie, *P* values).

### Recruitment

Participants will be recruited from metropolitan, suburban, and rural regions in New Mexico to ensure representation of families from diverse geographic, ethnic, and socioeconomic backgrounds. Recruitment will use a multimodal strategy that includes an informational study website, targeted online advertising, outreach to local autism support networks, and distribution of printed materials in community settings. Partnerships with pediatric and behavioral health providers will support referral-based recruitment. All recruitment materials and advertisements will be reviewed and approved by the Stanford University IRB before use. Interested caregivers will be directed to a secure REDCap link to complete the preliminary screening survey.

### Allocation

#### Sequence Generation

Randomization will be conducted in REDCap using a randomization code with an allocation ratio of 1:1:1 and a block size of 6. The study statistician will generate the randomization code and provide the randomization schedule to an independent REDCap administrator, who will upload the randomization scheme into the secure REDCap randomization module.

#### Allocation Concealment Mechanism

Allocation concealment will be maintained through REDCap’s centralized randomization module, which prevents study personnel from accessing or predicting assignment prior to randomization. Because the randomization sequence is stored within the secure REDCap environment and controlled by an independent data manager, no physical allocation lists or files will be accessible to the research team. Each participant’s assignment will remain unknown until eligibility is confirmed and baseline assessments are substantially complete. This process will ensure that participant enrollment and intervention assignment remain temporally separated, prevent allocation bias, and support the timely initiation of services.

#### Implementation

Participants will be enrolled by the study coordinator, who will assign each caregiver-child dyad to an intervention arm using REDCap’s automated randomization feature.

#### Blinding

Because session format (telehealth vs in person) is visually apparent, blinding of participants, clinicians, and observers is not feasible. To minimize bias, observers will use standardized operational definitions, and all coders will complete extensive training and achieve ≥85% interobserver agreement (IOA) before coding. IOA will be monitored on at least 25% of coded sessions.

The study statistician and PIs will remain blinded to group allocation during data analysis. Analyses will be conducted on deidentified datasets containing only coded group labels (eg, groups A, B, and C), and the randomization key will remain with the independent REDCap administrator until all analyses are complete. Although blinding is not feasible, these procedures will minimize the risk of bias and preserve analytic integrity. Because participants, clinicians, and observers are not blinded, an unblinding procedure is not applicable. The analytic team will remain blinded to group assignment until all analyses are complete.

#### Data Collection Methods

Participants will complete questionnaires at baseline (T0), 4 weeks (T4), 8 weeks (T8), 12 weeks (T12), and at 16 weeks (4-week posttreatment follow-up [T16]). Clinical trial staff, under the supervision of the PI and site-PI, will collect all questionnaire and observational data. All clinical data will be entered directly into REDCap from the source documents by authorized study personnel to maintain data integrity.

#### Interobserver Agreement

To ensure the reliability of observational outcome measures, IOA will be calculated on 25% of all primary observation points (T0-T16). Trial-by-trial IOA will be calculated by dividing the number of agreements by the number of trials in a session block and then multiplying the quotient by 100. An agreement will be scored if both observers score the same number of behaviors in a trial. For caregiver and clinician fidelity measures, total-count IOA will be calculated by dividing the number of agreements by the total number of events in each session block and multiplying by 100. Additional session-level data collected for monitoring intervention progress will not undergo IOA scoring, as these data will not be included in the trial outcomes.

### Caregiver Fidelity of Implementation

Caregiver fidelity data will be collected during each session using a standardized checklist monitoring 3 types of events: child-led play (9 items), adult-led instruction (10 items), and transition events (5 items) (Table S1 in Multimedia Appendix 1). Fidelity will be calculated as the percentage of correctly implemented steps (number of correctly implemented steps divided by the total number of events and then multiplied by 100).

### Clinician Fidelity of Implementation

Clinician fidelity data will be collected in 10% of sessions using a standardized checklist monitoring the teaching process (Table S2 in Multimedia Appendix 1). Clinician fidelity data will be reported as the number of events correctly implemented divided by the total number of events and then multiplied by 100 to obtain the percent correct.

### Participant Retention

If a participant misses a scheduled visit, research staff will contact the caregiver, attempt to reschedule within 1 week, and emphasize the importance of maintaining the visit schedule. Up to 3 contact attempts (telephone, email, or text) will be made before a participant is considered lost to follow-up. All contact attempts will be documented in the participant’s study record.

Each caregiver-child dyad will be eligible to receive up to US $200 in honoraria for completing study assessments. US $50 gift cards will be distributed at baseline and weeks 4, 8, and 12. Families who withdraw before study completion will receive prorated compensation based on completed assessments. Participants who discontinue the intervention will not be asked to complete additional outcome measures; however, all data collected up to the point of withdrawal will be retained for analysis. Reasons for discontinuation will be documented to support transparent reporting of attrition.

### Data Management

All study data will be stored in REDCap, a secure, 21 CFR Part 11–compliant data capture system. REDCap includes automated validation checks to support data completeness and internal consistency. All data management systems used by the study team will be secured and password-protected. Upon completion of the study, all datasets will be deidentified and archived in accordance with institutional data retention policies and IRB requirements. Deidentified data may be shared for secondary analyses or replication requests, provided appropriate safeguards are in place and release is approved by the IRB.

### Confidentiality

All personally identifiable information will remain confidential and accessible only to authorized study personnel. Identifiers will be stored separately from research data and replaced with a unique study ID for analysis. Identifiable contact information will be stored securely within Stanford University systems. Video recordings and associated data will be stored on encrypted servers and destroyed at the end of the study in accordance with the approved data management plan and IRB requirements. Only deidentified data may be retained for dissemination or data sharing. No biological specimens will be collected or stored for this study.

### Statistical Methods

In this longitudinal randomized controlled trial (RCT), 90 caregiver-child dyads of children with ASD will be assessed repeatedly at baseline, weeks 4, 8, and 12, and at 4-week posttreatment follow-up, allowing for strong inference regarding the longitudinal relationships among behavioral treatment, treatment fidelity, and challenging behaviors. The core analysis strategy in this project is longitudinal mixed-effects modeling, fully using the repeatedly measured primary and secondary outcomes. For maximum likelihood estimation of mixed-effects models, Mplus will be used. Because caregivers’ fidelity in implementing the same treatment will be observed under both in-person and telehealth conditions, we will examine the mediating effect of treatment fidelity (rather than conducting per-protocol analyses). We will follow the eligibility and analytical criteria of the MacArthur approach to mediator analysis [60], which will be embedded in our mixed-effects models. Specifically, mediation will be examined through two tests: (1) whether treatment via telehealth or in person significantly increases caregiver fidelity (eligibility criterion for mediators) and (2) whether the fidelity is correlated with caregiver stress (analytical criterion for mediators). As part of the sensitivity analysis, we will also use modern causal mediation approaches that use potential outcomes and propensity scores.

We will first estimate the main effect of the intervention on our primary outcomes, the rate of child challenging behavior and caregiver stress, using standard mixed-effects modeling, fully using all available repeated measures (0-, 4-, 8-, 12-, and 16-week follow-up). The primary parameter of interest is the group difference at 12 weeks (end of treatment). We expect that children who receive telehealth-delivered SBT or in-person SBT will show significant decreases in challenging behavior compared with those who receive caregiver online psychoeducation. We further expect that caregivers who receive telehealth-delivered SBT will report significantly lower scores on the total score of the PSI-4-SF than those who receive in-person SBT.

Using the same analytical method, we will also examine several secondary caregiver outcomes, including quality of life and treatment satisfaction. A categorical secondary outcome, the number of family and provider cancellations, will be modeled fully using individual missing data status at all 5 assessments. For this analysis, we will use generalized mixed-effects modeling, treating missing data status as a repeatedly measured binary outcome. We will also estimate group differences at the 4-week posttreatment follow-up, as this will provide valuable insight for clinicians and families on the short term and sustained effects of caregiver-mediated behavioral therapy for challenging behavior in children diagnosed with ASD.

To examine heterogeneity in treatment effects, we will assess the child’s age and baseline level of adaptive behavior as potential moderators of the treatment effect. For this investigation, we will use the MacArthur framework for moderator analysis, following the eligibility and analytical criteria for determining moderators, which are also embedded in our longitudinal mixed-effects modeling. We will also explore other possible family- and provider-level sociodemographic moderators of treatment response.

No interim analyses are planned. Because this is a low-risk behavioral intervention trial with minimal anticipated AEs, formal interim analyses for harm, efficacy, or futility are not warranted. The study statistician will remain blinded to group allocation until all data are collected and the databases are locked.

### Protocol Deviations and Missing Data

This protocol defines a protocol deviation as any noncompliance with the clinical trial protocol, International Council for Harmonisation Good Clinical Practice, or Manual of Procedures requirements. Noncompliance may be attributable to the participant, the investigator, or the study site staff. As a result of deviations, corrective actions will be developed by the site and implemented promptly. It will be the responsibility of the site investigator to exercise continuous vigilance to identify and report deviations within 7 working days of their identification or within 14 working days of the scheduled protocol-required activity. All deviations will be addressed in the study source documents and reported to the Department of Defense program official. We will implement rigorous efforts to minimize missing data. Nonetheless, missing data may occur due to the follow-up period. Missing data due to attrition or incomplete assessments will be handled under the assumption that data are missing at random, conditional on observed information.

### Data Access and Sharing

Deidentified quantitative data (eg, caregiver survey scores and summary outcome measures) may be made available upon reasonable request and with IRB approval. Video-based material will not be publicly available to protect participant confidentiality.

### Oversight and Monitoring

The trial will be jointly coordinated by Stanford University and Behavior Change Institute. The PI (SSH) and site-PI (JJP) will provide overall scientific leadership and operational oversight, including protocol adherence, data integrity, and communication with the funding agency. A core coordinating team comprising the PI, site PI, site investigators, project coordinator, and data manager will meet regularly to review recruitment progress, data quality, and intervention fidelity.

Because this is a minimal-risk behavioral intervention with no pharmacologic or invasive procedures, a formal data safety monitoring board is not required. In accordance with Department of Defense requirements, a data and safety monitoring plan has been developed and will be implemented throughout the trial. The PI will conduct ongoing safety oversight in collaboration with the Stanford IRB and WIRB-Copernicus Group Independent Review Board (WCG IRB). All unanticipated problems, AEs, and protocol deviations will be promptly reviewed and reported in accordance with institutional and regulatory requirements.

Operational oversight will include weekly meetings among the core study team to monitor recruitment, participant progress, and protocol adherence. The full study team will meet twice weekly to review trial operations, identify emerging issues, and implement corrective actions as needed. Each site will maintain internal quality assurance procedures to ensure participant protection and accurate, complete data collection across all phases of the trial.

### Adverse Events

This protocol defines an adverse event (AE) as any event that may impact a participant’s daily activities or functioning. AEs will be graded by severity (mild, moderate, and severe), relatedness (not related, unlikely, possibly, probably, and definitely), and action taken (none, discontinued temporarily, and discontinued permanently).

All AEs will be assessed by a member of the core study team in consultation with the caregiver to determine their relationship to study procedures. AEs will be documented in REDCap and followed until resolution or stabilization. Any medical or psychiatric condition present at screening will be considered baseline and not reported as an AE unless the condition worsens during the study, in which case it will be recorded as an AE. Changes in the severity or duration of any AE will also be documented.

At each study visit, the investigator will inquire about the occurrence of AEs or serious adverse events (SAEs) since the prior visit. An SAE is defined as a life-threatening event that results in death, hospitalization, disability, or permanent damage, or requires intervention to prevent permanent impairment. SAEs may come to the attention of study personnel during visits, caregiver reports, or routine monitoring.

All AEs and SAEs will be reviewed to determine potential relevance to the study. SAEs will be reported to the Stanford IRB within 24 hours of awareness. A trained member of the study team, in consultation with the PI, will evaluate each SAE, and a complete report will be submitted to the IRB no later than 10 working days after initial notification.

### Auditing

No formal or independent audits are planned due to the study’s minimal risk. The PIs and IRB will periodically review trial conduct. If required, the PI will provide direct access to all trial-related sites, source data and documents, and reports for monitoring and auditing by the funding agency and for inspection by local or regulatory authorities.

## Results

The study was funded in May 2022. Participant recruitment began in January 2024 and is projected to conclude in October 2026. As of March 2026, 81 caregiver-child dyads have been enrolled, with equal allocation across the 3 study groups (27 per group; 33%). Data analysis is expected to occur between Winter 2026 and Spring 2027, with primary results anticipated in Fall 2027.

## Discussion

### Anticipated Findings

This randomized trial directly compares telehealth and in-person delivery models to generate rigorous evidence regarding the effectiveness and feasibility of caregiver-mediated behavioral interventions for addressing challenging behaviors in children with ASD. The primary aim of this study is to determine whether caregiver-mediated behavioral treatment delivered via telehealth or in person results in greater reductions in child challenging behavior and caregiver stress compared with caregiver psychoeducation alone. We hypothesize that caregiver-mediated behavioral treatment delivered either via telehealth or in person will lead to greater reductions in child challenging behavior compared with caregiver psychoeducation alone. We also anticipate that telehealth delivery may produce comparable or greater reductions in caregiver stress relative to in-person delivery due to increased convenience, reduced logistical burden, and greater integration of intervention strategies within natural home environments.

### Comparison With Prior Work

Previous research has demonstrated that caregiver-mediated behavioral treatment can effectively reduce challenging behavior in children with ASD and improve caregiver outcomes. Telehealth delivery models have shown promise in increasing accessibility and reducing barriers to treatment, particularly for families in rural or underserved regions. However, few studies have directly compared telehealth and in-person delivery models for behavioral interventions targeting challenging behavior within randomized designs. This study builds on prior work by evaluating these delivery modalities within a rigorous randomized framework while incorporating community-informed outcomes that reflect the priorities of families and payers.

Beyond the scientific literature, there is a critical need for data that help families, providers, and payers make informed decisions about behavioral care for children with ASD. Insurance partners from our community advisory board have noted that the limited number of RCTs and comparative effectiveness research remains a barrier to broader coverage of telehealth-delivered ABA services. Reflecting the priorities of our ASD community advisory board, this trial prioritizes outcomes, such as caregiver stress and family quality of life, that extend beyond clinical symptom reduction to capture the broader impact of intervention on families.

### Strengths and Limitations

This study has several strengths. First, the RCT design provides a rigorous method for evaluating the comparative effectiveness of telehealth and in-person caregiver-mediated behavioral treatment. Second, the study incorporates community engagement throughout the design process, ensuring that the outcomes assessed reflect priorities identified by caregivers, clinicians, and payer representatives. Third, the intervention is delivered in the family home environment, increasing ecological validity and supporting the development of sustainable caregiver-implemented strategies.

Several limitations should also be acknowledged. This study is conducted at a single research site, which may limit the generalizability of the findings to other clinical settings or service delivery systems. In addition, participation is limited to English-speaking caregivers, which may reduce the applicability of findings to linguistically diverse populations. Additionally, the study excludes children who engage in the most severe forms of dangerous behavior that may require medical intervention or intensive clinical supervision. As a result, the findings may not fully generalize to families of children with the highest levels of behavioral risk, who may require more intensive or specialized services.

### Future Directions and Conclusions

If the intervention demonstrates positive outcomes, future research could extend this model toward tiered service-delivery approaches that integrate both caregiver-mediated and provider-delivered ABA treatment. Such frameworks could expand access to evidence-based care while reducing costs and family burden, aligning with modern standards of trauma-informed, family-centered behavioral health. Ultimately, this project aims to generate high-quality evidence supporting the responsible and equitable expansion of telehealth services for families of children with ASD. Future studies may also examine strategies to adapt and extend telehealth-delivered caregiver coaching models to linguistically diverse populations, and to children with more complex or severe behavioral presentations. In addition, future research may evaluate the long-term sustainability of caregiver-mediated interventions and their impact on service utilization, cost of care, and family well-being.

Although many funders now cover telehealth [61], the continued expansion of these services will be strengthened by rigorous, controlled evidence demonstrating clinical effectiveness and family benefit. This trial will provide comparative effectiveness data that may inform payer policy, clinical decision-making, and equitable service delivery. In addition, by empowering caregivers as primary agents of change within natural home environments, this model may support more sustainable, long-term behavioral solutions, particularly for families in underserved regions and those encountering systemic barriers to timely care.

### Dissemination Plan

Upon the successful completion of this clinical trial, the treatment manual will be made publicly available on the study website to support the dissemination of compassionate, trauma-informed SBT procedures. Study findings will be disseminated through peer-reviewed publications and presentations at national and international conferences focused on ASD research and behavioral health. Authorship eligibility will be determined in accordance with the International Committee of Medical Journal Editors guidelines. Results will also be shared with community partners and caregiver organizations to support the translation of findings into practice.

## Supplementary material

10.2196/91840 Multimedia Appendix 1 Skill-based treatment caregiver and clinician fidelity checklists.

10.2196/91840 Peer Review Report 1 U.S. Army Medical Research and Development Command Congressionally Directed Medical Research Programs Fiscal Year 2021, Autism Research Program Review Committee (Department of Defense, USA).

## Abbreviations

ABA: applied behavior analysis
AE: adverse event
APSI: Autism Parenting Stress Index
ASD: autism spectrum disorder
BPI-S: Behavior Problems Inventory—Short Form
BTPS-exp: Barriers to Treatment Participation Scale—Expectancies
CAB: contextually appropriate behavior
CARS-2: Childhood Autism Rating Scale, 2nd Edition
EO: establishing operation
FCT: functional communication training
HRE: happy, relaxed and engaged
IISCA: interview-informed synthesized contingency analysis
IOA: interobserver agreement
IRB: institutional review board
PedsQL: Pediatric Quality of Life
PFA: practical functional assessment
PI: principal investigator
PMI: Parent Motivation Inventory
PSI-4-SF: Parenting Stress Index—Fourth Edition Short Form
RCT: randomized controlled trial
REDCap: Research Electronic Data Capture
SAE: serious adverse event
SBT: skill-based treatment
sFCR: simple functional communication response
SPIRIT: Standard Protocol Items: Recommendations for Interventional Trials
SR: synthesized reinforcement
SRS-2: Social Responsiveness Scale, Second Edition
TARF-R: Treatment Acceptability Rating Form–Revised
Vineland-3: Vineland Adaptive Behavior Scales, Third Edition
