# Supplementary material for: Telehealth Versus In-Person Caregiver-Mediated Behavioral Treatment for Challenging Behaviors in Children With Autism Spectrum Disorder: Protocol for COACH (Caregiver Outreach for Autism Coaching at Home) Randomized Controlled Trial
Source: JMIR Res Protoc. 2026 May 26;15:e91840. doi: 10.2196/91840 (PMC13211944; doi:10.2196/91840)
Supplement: Multimedia Appendix 1 [file resprot-v15-e91840-s001.docx]

**SBT Caregiver Fidelity Checklist**

**Treatment Step:** ☐ sFCR ☐ cFCR ☐ CAB1 ☐ CAB2 ☐ CAB3

**Implementor:___________________________ Observer:____________________________ Date:____________________**

|  |  | Trial 1 | Trial 2 | Trial 3 | Trial 4 | Trial 5 |
| --- | --- | --- | --- | --- | --- | --- |
| ***Child-Led Time*** | | | | |  |  |
| **DO’s** |  |  |  |  |  |  |
| - | Caregiver had many of the learner's preferred items/activities available. |  |  |  |  |  |
| - | Caregiver was available to and engaged with the child (close in proximity, not distracted, and providing high quality attention in the manner the child prefers). |  |  |  |  |  |
| - | Caregiver stayed in child-led time until the child was happy, relaxed, and engaged for at least 30 seconds. |  |  |  |  |  |
| **-** | If the child made a request: Caregiver honored all reasonable requests for items, attention, or saying/doing things a particular way. |  |  |  |  |  |
| **-** | If the child made an unreasonable request: Caregiver denied the unreasonable request and empathized. Caregiver non-verbally made sure other items were available. |  |  |  |  |  |
| **-** | If challenging behavior occurred: Caregiver responded by acknowledging it (e.g., made an empathic statement) and made a change (removed any unplanned EOs). |  |  |  |  |  |
| **DON’Ts** |  |  |  |  |  |  |
| - | Caregiver refrained from placing any demands, including instructions and questions (i.e., made it clear that the child was in charge and caregiver was following their lead). |  |  |  |  |  |
| - | Caregiver refrained from correcting the child (including providing feedback on past challenging behavior) or the way they were engaging with an item/activity. |  |  |  |  |  |
| - | Caregiver did not attempt to redirect or verbally offer choices following denial of unreasonable requests or following challenging behavior. |  |  |  |  |  |
|  |  |  |  |  |  |  |
|  | **If caregiver interacted correctly (all DO’s and DON’Ts), check box:** | ☐ | ☐ | ☐ | ☐ | ☐ |
|  |  |  |  |  |  |  |
|  |  | Trial 1 | Trial 2 | Trial 3 | Trial 4 | Trial 5 |
| ***Adult-Led Time*** | | | | |  |  |
| **DO’s** |  |  |  |  |  |  |
| - | Caregiver delivered clear cues to signal the change to adult-led time, progressively in a stepwise manner. |  |  |  |  |  |
| - | Caregiver used clear, concise instructions (e.g., playtime is all done). |  |  |  |  |  |
| - | Following instruction, caregiver delivered a prompt for the target response (e.g., "my way, please") or moved through a prompt sequence as needed. |  |  |  |  |  |
| - | Caregiver only allowed access to materials relevant to what the child was expected to do. |  |  |  |  |  |
| - | If challenging behavior occurred, caregiver remained neutral and prompted the child to engage in the CAB response, unless a safety concern was indicated. |  |  |  |  |  |
| **DON’Ts** |  |  |  |  |  |  |
| - | Caregiver refrained from presenting instructions as questions or options. |  |  |  |  |  |
| - | Caregiver refrained from complying with child attempts to lead instruction (e.g., "I want to clean up before I sit at the table"). |  |  |  |  |  |
| - | Caregiver refrained from negotiating, arguing, rationalizing, cajoling, or repeating prompts that have been ineffective. |  |  |  |  |  |
| - | Caregiver did not react in any obvious way to inappropriate behavior and simply proceeded with the 3-step prompting or agreed upon alternative prompt. |  |  |  |  |  |
| - | Caregiver refrained from changing the demand contingent on challenging behavior. |  |  |  |  |  |
|  |  |  |  |  |  |  |
|  | **If caregiver interacted correctly (all DO’s and DON’Ts), check box:** | ☐ | ☐ | ☐ | ☐ | ☐ |
|  |  |  |  |  |  |  |
|  |  |  |  |  |  |  |
|  |  |  |  |  |  |  |
|  |  |  |  |  |  |  |
|  |  | Trial 1 | Trial 2 | Trial 3 | Trial 4 | Trial 5 |
| ***Transition from Adult-Led to Child-Led Time*** | | | | |  |  |
| **DO’s** |  |  |  |  |  |  |
| - | Caregiver delivered clear cues to signal return to child-led time, all at once (not in a stepwise manner). |  |  |  |  |  |
| - | Caregiver moved from adult-led time to child-led time only after the child demonstrated an appropriate skill (e.g., communication or CAB). |  |  |  |  |  |
| - | If teaching contextually appropriate behavior (CAB), caregiver sometimes rewarded functional communication skills. |  |  |  |  |  |
| **DON’Ts** |  |  |  |  |  |  |
| - | If challenging behavior occurred, caregiver refrained from habitually reinforcing it. If reinforcement of challenging behavior was necessary, caregiver reflected and made changes prior to the next trial. |  |  |  |  |  |
| - | Caregiver refrained from foreshadowing which skills would be reinforced or how many demands would need to be completed prior to earning child-led time (i.e., kept it unpredictable). |  |  |  |  |  |
|  | **If caregiver interacted correctly (all DO’s and DON’Ts), check box:** | ☐ | ☐ | ☐ | ☐ | ☐ |
|  | | | | |  |  |

**Note.** Fidelity scored on a trial-by-trial basis. Adapted from *Practical Functional Assessment and Skill-Based Treatment Workbook* (FTF Behavioral Consulting, [www.ftfbc.com](http://www.ftfbc.com)).

**SBT Clinician Fidelity Checklist**

Participant #: _______________ Coder: _______________ Date: _______________

Phase: ☐ sFCR ☐ cFCR ☐ CAB1 ☐ CAB2 ☐ CAB3 New Step: ☐ Yes ☐ No

|  |  | **Yes** | **No** | **NA** |  |
| --- | --- | --- | --- | --- | --- |
| ***Teaching New Steps/Retraining (If this is a new step, begin with #1; if not, begin with #6 and score items 1–5 as NA.)*** | | | | | |
| 1 | Clinician introduces and reviews the new SBT step with the caregiver. | ☐ | ☐ | ☐ |  |
| 2 | Clinician provides rationale for new step. | ☐ | ☐ | ☐ |  |
| 3 | Clinician provides verbal or physical models specific to the new step. | ☐ | ☐ | ☐ |  |
| 4 | Clinician role plays with caregiver as needed and provides feedback as needed. | ☐ | ☐ | ☐ |  |
| 5 | Clinician guides the caregiver to practice the new SBT step with the child, providing prompts as needed. | ☐ | ☐ | ☐ |  |
| ***During Teaching Process*** | | | | | |
| 6 | Clinician reviews the current step at the beginning of the visit (score NA if new step). | ☐ | ☐ | ☐ |  |
| 7 | At the beginning of the visit, clinician coaches the caregiver to have the child’s preferred reinforcers available for a minimum of 4 minutes of child-led play, and minimize evocative events such as giving instructions or asking questions. | ☐ | ☐ |  |  |
| 8 | Clinician guides the caregiver to conduct 5 trial blocks appropriate to the step (e.g., if CAB step, the clinician coaches caregiver to use a mixed trial format). | ☐ | ☐ |  |  |
| 9 | Clinician guides the caregiver to deliver the EO specific to the SBT step. | ☐ | ☐ |  |  |
| 10 | Clinician guides the caregiver to deliver reinforcement after child demonstrates the correct response specific to the SBT step. | ☐ | ☐ |  |  |
| 11 | Clinician waits until the child is happy, relaxed and engaged for a minimum of 30 seconds before coaching the caregiver to implement the next trial. | ☐ | ☐ |  |  |
| 12 | Clinician gave specific praise to the caregiver for implementing the correct step (e.g., correct prompt type, reinforcement timing, response to sFCR/cFCR). | ☐ | ☐ |  |  |
| 13 | Clinician provides corrective feedback to the caregiver on incorrect implementation and/or omissions for the current SBT step. (If not applicable, score NA.) | ☐ | ☐ | ☐ |  |
| 14 | Clinician provides opportunities for the caregiver to ask questions throughout. | ☐ | ☐ |  |  |
| ***End of Visit Procedures*** | | | | | |
| 15 | Clinician ends the session with the child being happy, relaxed and engaged, and not displaying problem behavior before closing out the visit. | ☐ | ☐ |  |  |
| 16 | Clinician debriefs the caregiver at the end of the visit, reviewing what occurred during the session and discussing next steps as needed. | ☐ | ☐ |  |  |
|  | | | | | |

# Items Scored Yes: _______ Total Items Scored (Yes and No): _______ % Fidelity: _______
