# Supplementary material for: Telehealth Versus In-Person Caregiver-Mediated Behavioral Treatment for Challenging Behaviors in Children With Autism Spectrum Disorder: Protocol for COACH (Caregiver Outreach for Autism Coaching at Home) Randomized Controlled Trial
Source: JMIR Res Protoc. 2026 May 26;15:e91840. doi: 10.2196/91840 (PMC13211944; doi:10.2196/91840)
Supplement: Peer Review Report 1 [file resprot-v15-e91840-s002.pdf]

Department of Defense
U.S. Army Medical Research and Development Command
Congressionally Directed Medical Research Programs
Fiscal Year 2021 Autism Research Program
Clinical Trial Award
Peer Review Summary Statement

CDMRP Log Number: AR210221
Grants.gov ID Number: GRANT13439477
Review Panel: Clinical Translational Research - Clinical Trial - 2
Discussion Period: 09/28/2021-09/29/2021

Project Duration: 48 months
Budget Requested: $2,261,316
Direct Costs: $1,796,044
Indirect Costs: $465,272

Title: Randomized Controlled Trial of Telehealth-Enabled Versus In-Person Parent-Mediated Behavioral Treatment for Challenging Behaviors in Autism Spectrum Disorder
Principal Investigator: Scott Hall
Performing Organization: Leland Stanford Junior University, The
Contracting Organization: Leland Stanford Junior University, The

OVERVIEW

The Principal Investigator (PI) of this application proposes to conduct a more rigorous assessment of the relative strengths and benefits of remote-based versus in-person delivery of parent-mediated behavioral therapy using a randomized controlled trial (RCT) design. The specific aims are (1) to conduct qualitative interviews with caregivers, treatment providers, and insurance stakeholders, in partnership with a caregiver and provider advisory board, to define the needs and barriers to providing parent-mediated interventions for challenging behaviors in children diagnosed with autism spectrum disorder (ASD) via telehealth and (2) to conduct an RCT to compare telehealth versus in-person delivery of parent-mediated behavioral therapy based on the principles of applied behavioral analysis (ABA) and designed specifically for the treatment of challenging behaviors in children diagnosed with ASD. A 3-group RCT will be performed. The intervention is parent-mediated behavioral therapy based on the principles of ABA, and the projected results are child problem behavior will be significantly lower post treatment for the remote and in-person parent-mediated treatment groups compared to the control group, and parent stress will be significantly lower post treatment for the remote parent-mediated treatment group than the in-person or control groups.

Procurement Sensitive Document
Do not copy or distribute without CDMRP written permission.
Prepared by SRA International, Inc., A GDIT Company, a support contractor to CDMRP

	Average Score	Standard Deviation
**Overall Evaluation**<br>*Rating Scale: 1.0 (highest merit) to 5.0 (lowest merit)*	1.6<br>**(Excellent)**	0.2

**Criteria**<br>*Rating Scale: 10 (highest merit) to 1 (lowest merit)*	**Average Score**
**Scientific Merit**	8.0
**Clinical Impact**	7.6
**Statistical Plan**	9.1
**Intervention**	8.3
**Recruitment, Accrual, and Feasibility**	7.2
**Ethical Considerations**	8.6
**Personnel and Communication**	8.2
**Regulatory Strategy and Transition Plan**	8.6

SCORED CRITERIA

Scientific Merit
Average Score: 8.0

Scientist Reviewer A

This study will be conducted as an RCT. Initial stakeholder input on needs and barriers related to parent-mediated interventions for challenging behavior with children diagnosed with ASD will be used to enhance real-world applicability of the intervention to be tested. Following screening for inclusion (eg, confirmation of ASD diagnosis, child age, daily occurrence of challenging behavior) and consent, baseline assessment using functional analysis of problem behavior and parent rating scales (measuring parent stress, child adaptive behavior, family quality of life) will be conducted for 180 parent-child dyads. Participants will then be randomly assigned to 1 of 3 conditions: remote parent-mediated behavioral treatment, in-person parent-mediated behavioral treatment, or only psychoeducation (control). Participants assigned to the remote treatment condition will receive 60-minute therapy sessions weekly for 12 weeks, using a function-based treatment informed by the functional analysis results. Participants in the in-person treatment condition will receive the same approach as those in the remote treatment condition, but all sessions will be conducted in a clinic setting. In the control condition, parent participants will be required to view online training videos on using behavioral techniques for children with ASD. These online videos will be supplemented with 20- to 40-minute meetings (to match the session time in other conditions) with research assistants to discuss experiences using the training videos. Participants in each condition will complete ratings scale assessments at baseline, midtreatment (Weeks 4 and 8), post treatment (Week 12), and 12 weeks post treatment (Week 24). Based on power analysis with consideration for 20% attrition, 180 parent-child dyads are indicated. Primary and secondary outcome data will be evaluated using mixed-effects modeling.

Strengths: This proposal is based on solid preliminary data from previous research conducted by this team with a similar population (ie, fragile X) and decades of research supporting function-based behavioral treatments for children with ASD exhibiting challenging behavior. Most published studies of behavior-analytic treatments for problem behavior utilize single-case design. Thus, large sample size studies of function-based behavioral treatments like the proposed study are needed to establish effectiveness and efficiency of these treatments. The aims and hypotheses are likely sufficient to answer the stated goals of

this study (ie, whether parent-mediated function-based treatments delivered via telehealth are superior to other treatment and as effective as in vivo delivery). The use of a psychoeducation control seems appropriate given that a large proportion of children with ASD are receiving insufficient intervention. The inclusionary and exclusionary criteria are well developed, reasonable, and based on previous successful research.

Weaknesses: There is a fairly robust research literature showing that in-person function-based therapy and telehealth-delivered function-based therapy are effective approaches to reducing challenging behavior in young children with autism. Although few RCTs have been conducted comparing in-person and telehealth-based behavioral treatment, the need to establish function-based treatment for challenging behavior as scientifically validated is unnecessary and may not add much to the current literature. Random assignment following the baseline functional analysis does not control for complexity of cases, and allocation bias is possible. Aim 1 does not link well with Aim 2.

Scientist Reviewer B

Strengths: The researchers offer a strong rationale for the proposed clinical trial given the high prevalence of challenging behavior among children diagnosed with autism and frequent challenges finding and accessing appropriate behavioral care. The PI has relevant completed and pending clinical trials in the treatment of disruptive behaviors and presents compelling preliminary data on 60 children with fragile X and disruptive behavior from a completed RCT comparing telehealth behavioral consultation versus treatment as usual. The inclusion of a stakeholder advisory board for Aim 1 ensures that treatment approach is codeveloped with input from families, teachers, clinicians, insurance payers, and professional association stakeholders. The proposed prospective RCT design has 3 arms to be able to compare the intervention of interest (telehealth parent-mediated behavior therapy) to 2 active contrast conditions (both in-person parent-mediated behavior therapy and online psychoeducation behavioral intervention modules) that are comparable in terms of intervention intensity and length. Data collection procedures and analyses are clearly described in great detail. Measures used in the proposed work are well-established, validated, and reliable tools that are serially administered to participated at multiple time points over the course of intervention. In addition to measures that capture reductions in disruptive behavior, the researchers include measures to capture some of the unique anticipated benefits potentially associated with a telehealth model (numbers of cancellations by families and clinicians, measures of parent stress and child anxiety). The analytic plan includes repeated measurement time points for both primary and secondary measures.

Weaknesses: There are existing published trials of telehealth and in-person parent-mediated behavior therapy, reducing the necessity and scientific rationale of the currently proposed trial.

Clinical Impact
Average Score: 7.6

Scientist Reviewer A

Strengths: The proposed study addresses 2 of the FY21 Autism Research Program (ARP) Clinical Trial Award areas of interest: (1) behavioral, cognitive, and other nonpharmacological therapies for ASD core symptoms or to alleviate co-occurring conditions (challenging behavior) and (2) dissemination/implementation of clinically validated treatments. This study addresses these areas by providing new information regarding predictors of function-based treatment success and the impact of different behavioral treatment types (eg, function-based versus psychoeducation) and modalities (in vivo versus telehealth) on problem behavior, parent stress, and quality of life. Although this treatment approach is not novel, limited research exists on the impact on parent stress and quality of life. The

Procurement Sensitive Document
Do not copy or distribute without CDMRP written permission.

proposal includes a commitment to disseminate findings across many channels, including professional/scientific conferences, peer-reviewed publication, and practitioner training via Project Extension for Community Healthcare Outcomes (ECHO). Of particular importance is reaching practitioners who may be reluctant to use telehealth delivery models. The sample population in this study (ie, young children with ASD and disruptive behavior) is an appropriate target for this study as effective early treatment is needed to prevent more deleterious outcomes likely to occur with untreated older children (ie, improve quality of life long term). Possible long-term benefits include an appealing option for service providers who would like to serve families living outside their service area and those with frequent relocations (eg, military families), reduced costs and time in travel for families and insurers, and greater maintenance of treatment effects due to parent-mediated delivery.

Weaknesses: Outcomes of this study are unlikely to decrease access delays to therapy (sometimes months or years) as there is a dearth of providers. Although telehealth may increase access to those in geographic areas without providers as telehealth can reach virtually any location, overall, it is unlikely to decrease wait times for families desiring services. Should those in underserved areas access telehealth-based intervention, it may have the unintended consequence of increasing wait times for those in geographic areas where in-person services currently exist. The proposed methods do not make mention of plans to actively recruit parent-child dyads of racial and ethnic minority groups, and the recruitment plan for families from geographically remote/rural areas as well as those without permanence or in the military is not very strong. The impact of the results may be considered less meaningful without inclusion of these groups.

Scientist Reviewer B

Strengths: This proposal addresses 2 ARP CTA areas of interest: dissemination/implementation of clinically validated interventions and behavioral, cognitive, and other nonpharmacological therapies for ASD core symptoms or to alleviate co-occurring conditions. Aggression and other significant disruptive behavior are common in autism and detrimental to individual and family quality of life. Long-term benefits are substantial as minimizing or eliminating disruptive behavior early in children’s lives facilitates their ability to live independently, receive and benefit from educational and therapeutic supports, build family connections, and maintain positive social relationships. This trial evaluates a telehealth-delivered parent-mediated behavior intervention with the goal of justifying and securing insurance coverage for telehealth behavioral services beyond COVID-19 temporary authorizations. The sample population will be drawn from 2 diverse states and is likely to be generalizable to other English-speaking individuals with autism and disruptive behavior.

Weaknesses: Non-English-speaking families will be excluded, and it is unclear how recruitment of underserved ethnic minority populations will be conducted.

Consumer Reviewer

Strengths: The realization of this proposal would help alleviate one of the most common complaints from caregivers, which is the lack of available and affordable ABA therapy. Often, ABA is one of the first techniques recommended to families, and when they are dealing with a novel diagnosis, many are desperate to begin but quickly encounter roadblocks with insurance denials, affordability, and then available therapists. ABA is often long term, and while it has been shown to sometimes eradicate undesirable behaviors, it is not translational. While this would not be the same as ABA, it would allow families to gain insight and trial behavioral techniques at home, lessening the need for the all-encompassing, expensive, long-term, traditional behavioral therapy. While the treatment of “behavioral symptoms” with behavioral therapy will not likely be largely impactful itself, alleviating the current challenges of access to ABA with telehealth options would definitely be positive, as would giving parents

Procurement Sensitive Document
Do not copy or distribute without CDMRP written permission.

short-term tools to deal with challenging behaviors. In addition, the long-term impact of providing critical data to support other types of telehealth care in ASD has significant potential.

Weaknesses: Differentiation in family dynamics, schedules, and parents' ability to effectively implement these techniques calls into question the validity of results. Also, this would not be accessible to families who are not fluent in English.

Statistical Plan
Average Score: 9.1

Scientist Reviewer A

Strengths: The statistical plan appears sufficient to answer the research questions of interest.

Weaknesses: The power analysis was conducted with an estimate of no more than 20% attrition, which was based on their pilot study and may not be adequate for this study. It is unclear why the application does not use direct observation data as a primary outcome measure in addition to the rating scales used to measure child behavior change.

Scientist Reviewer B

Strengths: Thematic analysis of stakeholder interviews will be facilitated by NVivo. The use of longitudinal mixed-effects modeling with multiple assessment time points is a significant strength as it allows for understanding the unique growth curves of each outcome.

Weaknesses: No weaknesses were noted.

Biostatistician Reviewer

Strengths: The study has provided preliminary data relevant to the proposed trial. The design of the RCT is appropriate. The rationales of sample size determination and power analysis are adequately presented. A reasonable attrition rate is considered in the trial design. In general, the statistical plan is well described. From data preprocessing (check for normality and consideration of transformation) to the choice of statistical methods (nonparametric tests, longitudinal mixed-effects model, handling of missing data, sensitivity analysis, etc), the data analysis plan should be able to address the research questions.

Weaknesses: No weaknesses were noted.

Intervention
Average Score: 8.3

Scientist Reviewer A

Strengths: The intervention proposed (functional communication training) in this study has been scientifically validated across numerous studies, including a recent RCT via telehealth with this population. There are many potential benefits to in-home parent-mediated delivery, including greater generalization and maintenance of treatment effects and family comfortability and empowerment. Unfortunately, neither in-home nor telehealth-delivered treatment is easily accessible currently, and either modality would likely be above the standard of care for the average family with a child with ASD. The duration of the intervention seems appropriate and long enough to detect any differences across primary and secondary measures. There are several components likely to mitigate attrition, and the use of Behavior Capture to ensure practice between therapy visits is an enhancement not commonly seen in

Procurement Sensitive Document
Do not copy or distribute without CDMRP written permission.

standard practice. The value of this study is neither the treatment nor the modality delivered. Rather, the proposal indicates that there is a need to establish function-based treatments as scientifically validated through RCTs, which, in turn, will result in greater payer coverage and improve access to treatment for individuals with ASD and their families. Despite the focus on challenging behavior, this treatment is unlikely to pose risks to safety, especially with the age range included in this study.

Weaknesses: It is unclear what, if any, modifications will be made to the procedures outlined in the proposal resulting from the stakeholder meetings. A large body of research to support this intervention already exists, with recent research also indicating success via telehealth delivery. Although more evidence of the effectiveness of this intervention may increase the likelihood that payers will cover this intervention via telehealth, it does not address a considerable challenge to access, which is lack of service providers. This study is only focused on a very narrow age range of children with ASD despite the fact that this intervention has already been evaluated to a great extent with young children with ASD.

Scientist Reviewer B

Strengths: ABA is a well-established and validated approach for addressing problematic behavior in children with autism. The researchers present evidence and preexisting data from their lab and other labs that the proposed telehealth intervention compares favorably to in-person behavior intervention efforts. Behavioral procedures of each RCT arm are clearly described, and all include opportunities for families to converse with a live clinician. ABA is currently the standard of care for autism treatment, but community implementation of ABA varies significantly in quality depending on geographic location and service provider availability.

Weaknesses: It could be made clearer how the proposed parent-mediated ABA interventions differ from existing community-implemented ABA to clarify how the proposed intervention is novel and worth examining in the proposed trial.

Recruitment, Accrual, and Feasibility
Average Score: 7.2

Scientist Reviewer A

Strengths: Recruitment for Aim 1 of the study seems very feasible given the positions the PIs hold and access to the various stakeholders. The proposal indicates access to a large number of potential subjects for the RCT through the Stanford Child Psychiatry Outpatient Clinic and the Behavioral Change Institute. Additionally, one of the PIs is involved in several networks of service providers from which they may be able to recruit. The research team was successful in recruitment of a smaller number of subjects for another clinical trial, carried out over a shorter time frame and with less research coordination support. The investigators have a well-developed plan that includes incentives and reminders of appointments to mitigate excessive attrition.

Weaknesses: There are no specific plans for recruitment of underserved and/or minority participants or to ensure that the target enrollment figures for a representative sample based on the US 2020 Census are met. There is insufficient mention within the consent process where the intervention will be conducted for those assigned to the in-person therapy condition. The budget includes support for travel reimbursement, which suggests that families will be expected to travel to one of the investigators' clinics for treatment. This may impact families in that condition to a greater extent than in the other 2 ars of the study.

Procurement Sensitive Document
Do not copy or distribute without CDMRP written permission.

Scientist Reviewer B

Strengths: The applicant's home institution outpatient clinic at Stanford and the Behavior Change Institute serve large numbers of patients annually and will serve as recruitment pools. The PI describes multiple complementary recruitment strategies to protect against inadequate recruitment. The researchers mention efforts to recruit from underserved and ethnically diverse areas.

Weaknesses: The anticipated enrollment table incorrectly shows equal numbers of male and female participants and 360 total participants (instead of the proposed 180). Additional detail is needed on specific methods the researchers will use to successfully recruit from underserved and ethnically diverse areas.

Ethical Considerations
Average Score: 8.6

Scientist Reviewer A

Strengths: The population selected for inclusion in this study is likely to benefit from the knowledge gained. The investigators make the case for indirect long-term benefits that may result from payer adoption of coverage for telehealth behavioral treatment based on evidence of scientific validation of this approach. The plan to minimize risk seems appropriate and well developed. The data safety monitoring board (DSMB) procedures are sound. Given the level of risk associated with this study, the investigators have developed a sufficient plan to monitor, report, and ameliorate any adverse events. Confidentiality and privacy are adequately addressed in the proposal.

Weaknesses: Although the 3-person DSMB may be capable of providing oversight of safety risks and progress toward scientific goals, it will be important to ensure independence. The 3 members composing the DSMB are colleagues of investigators on the research team, which can sometimes challenge independence. The DSMB should consider a biostatistician with expertise in clinical trial data and a community member or caregiver of an individual with ASD to evaluate the participant experience.

Scientist Reviewer B

Strengths: Human subject risks and protections/safeguards to physical safety, confidentiality, privacy, and autonomy are well described.

Weaknesses: No weaknesses were noted.

Bioethicist Reviewer

Strengths: Subjects of this study stand to benefit from the knowledge gained. The study procedures seem consistent with sound research design, and the procedures are in use for other treatment purposes. The consent process, maintenance of confidentiality, and protection of privacy are well described. Safeguards are in place for vulnerable populations. Risks are well described with measures in place to minimize risk.

Weaknesses: The applicants do not sufficiently consider how issues related to subject withdrawal/dropout will be managed (ie, when a child does not want to participate but the parent does).

Personnel and Communication
Average Score: 8.2

Procurement Sensitive Document
Do not copy or distribute without CDMRP written permission.

Scientist Reviewer A

The PI, Scott Hall, received a PhD in psychology from the University of London in 1997. Dr Hall is currently a professor of psychiatry and behavioral sciences and director of the Translational Applied Behavior Analysis Laboratory at Stanford University.

Co-PI Joy Pollard received a PhD in disability disciplines and applied behavior analysis from Utah State University in 2012. Dr Pollard is the codirector of the Behavior Change Institute (New Mexico) and an adjunct faculty member in the Department of Psychiatry and Brain Sciences at Stanford University.

Biostatistician Booil Jo received a PhD in applied statistics from the University of California, Los Angeles (UCLA) in 1998. Dr Jo is an associate professor of psychiatry and behavioral sciences at Stanford University.

Strengths: The team assembled for this study has the experience and expertise to conduct the study. The percentage effort for team members seems appropriate given their respective duties. Dr Scott Hall has experience in conducting an RCT and has research grant funding at the federal level. Dr Joy Pollard has a strong national reputation for her leadership related to clinical services for children with autism. She seems to have less experience with federally funded research grants but has been a Co-PI or coinvestigator on several large research studies. Dr Hall and Dr Pollard have demonstrated success working together on previous grants.

Weaknesses: The communication plan is not fully detailed and does not make mention of how disagreements across the 2 sites will be handled.

Scientist Reviewer B

Strengths: The team appears to have the relevant experience and expertise to complete the proposed investigation, having completed similar trials in the past.

Weaknesses: The communication plan requires additional detail to determine how project decisions will be carried out.

Regulatory Strategy and Transition Plan
Average Score: 8.6

Scientist Reviewer A

Strengths: The transition plan includes dissemination at multiple levels to reach professional and scientific audiences. The plan includes dissemination milestones that seem reasonable. There is a heavy emphasis on dissemination through the Project ECHO model, which has been effective in training providers in autism-specific assessment and treatment approaches using a relatively efficient model that utilizes teleconferencing.

Weaknesses: The transition plan timeline indicates only the roles of the PI and site PI in dissemination efforts, but it will likely require heavy support that is not made explicit in the plan.

Scientist Reviewer B

Strengths: The team plans to share project results through scientific conference presentation and peer-reviewed journal articles. The team plans to create practitioner training materials consisting of online webinars and a Project ECHO clinician mentorship model.

Weaknesses: No weaknesses were noted.

Technology Transfer Specialist Reviewer

Strengths: The next step identified by the application appears feasible—the project uses already-extant training materials and is simply looking at different delivery methods of those materials. The immediate knowledge product should be transferable without substantial barriers, and the next-step study identified appears to be development of further knowledge products. The regulatory plan is appropriately light as this has minimal regulatory oversight required. The proposed collaborations and resources appear to be a strength of this project, in particular, the proposed usage of the ECHO training module appears to be a significant strength. The schedule and milestones for further transition appear feasible and appropriate. There is a risk analysis provided, and it briefly addresses issues.

Weaknesses: The funding strategy for further development is light. While the proposal indicates minimal additional development is planned, it does not provide a funding strategy for that additional development. The risk analysis is brief and does not substantively identify or address likely risks (lack of uptake in the clinical community, lack of success in furthering coverage of this modality). The application does not appear to present an intellectual property (IP) plan. While it is likely that any IP generated (such as the training programs utilizing Project ECHO) will be governed by other terms and conditions and relevant IP policies, the application does not appear to provide any analysis of this. These are all likely minor weaknesses, however, given the nature of the project and the likely IP that may result.

UNSCORED CRITERIA

Environment

Scientist Reviewer A

The research environment at Stanford University is excellent and would provide much of the support needed to conduct this research study. Given that this team recently completed an RCT across their 2 sites, many details have been worked out for telehealth-based intervention; however, it is unclear what space will be available for conducting in-person therapy and how that space is configured. Institutional and collaborating agency letters of support strengthen this application.

Scientist Reviewer B

The institution appears well equipped to support the study.

Procurement Sensitive Document
Do not copy or distribute without CDMRP written permission.

Budget

Scientist Reviewer A

The direct costs associated with this proposal fall under the maximum allowable direct costs. The budget seems appropriate, but it is unclear whether additional costs will be incurred for use of clinic space at 1 of the 2 research sites.

Scientist Reviewer B

The budget looks appropriate for the research proposed.

Application Presentation

Scientist Reviewer A

The application is generally well written but with some details related to the study procedures for in-person therapy lacking. The timelines and tables were very helpful.

Scientist Reviewer B

The application is well written and organized with appropriate use of bold and underlined section headers.

Procurement Sensitive Document
Do not copy or distribute without CDMRP written permission.
